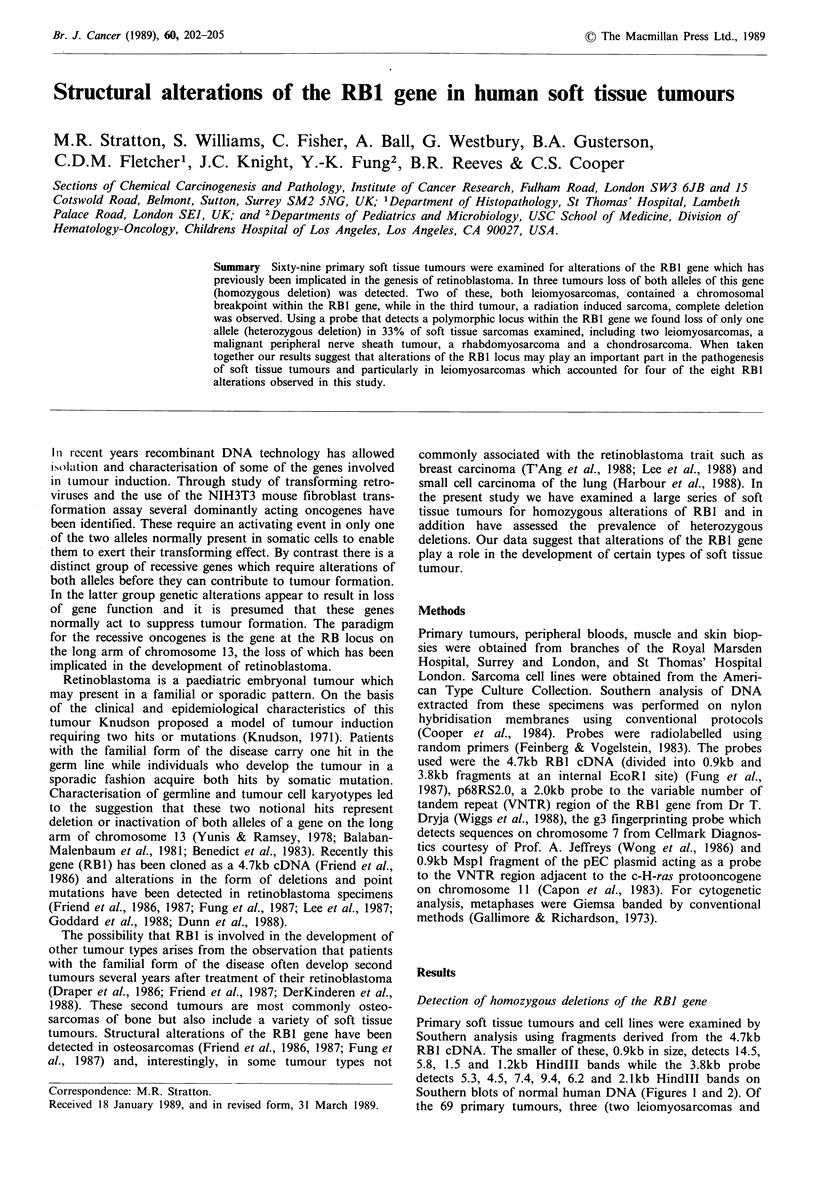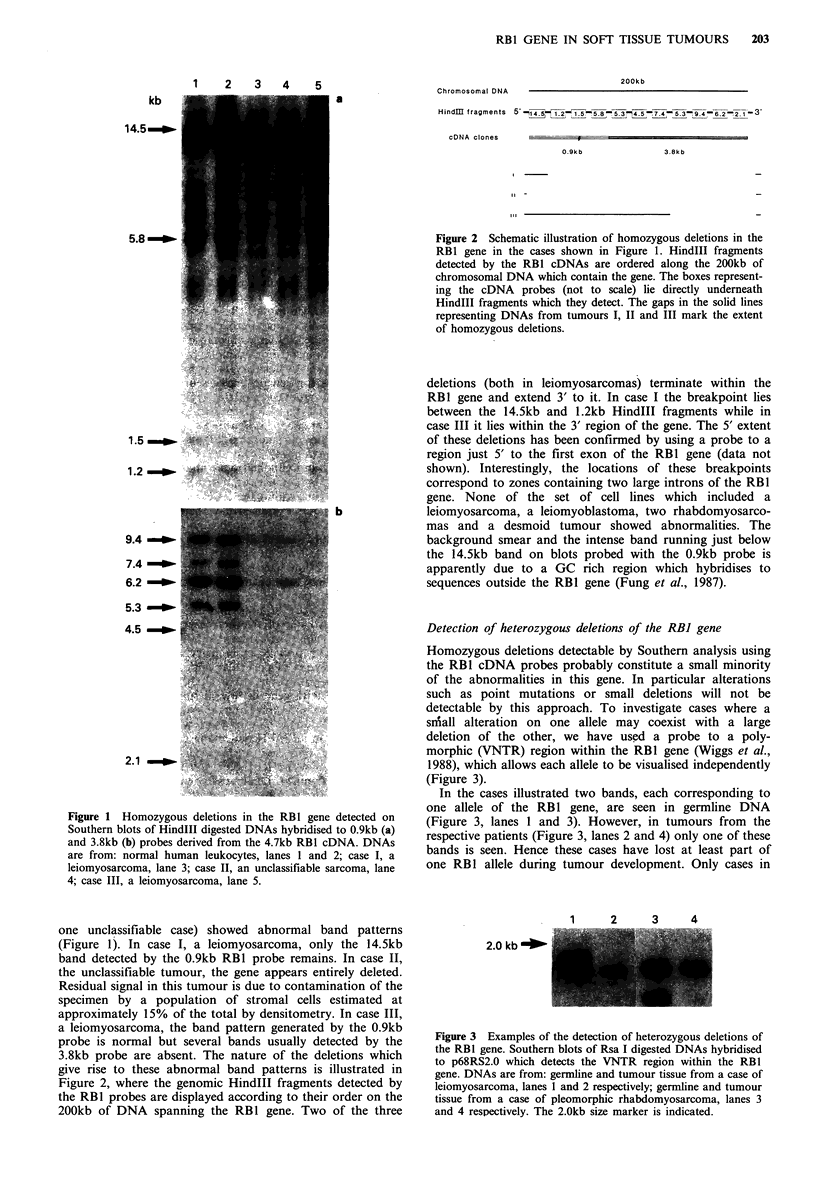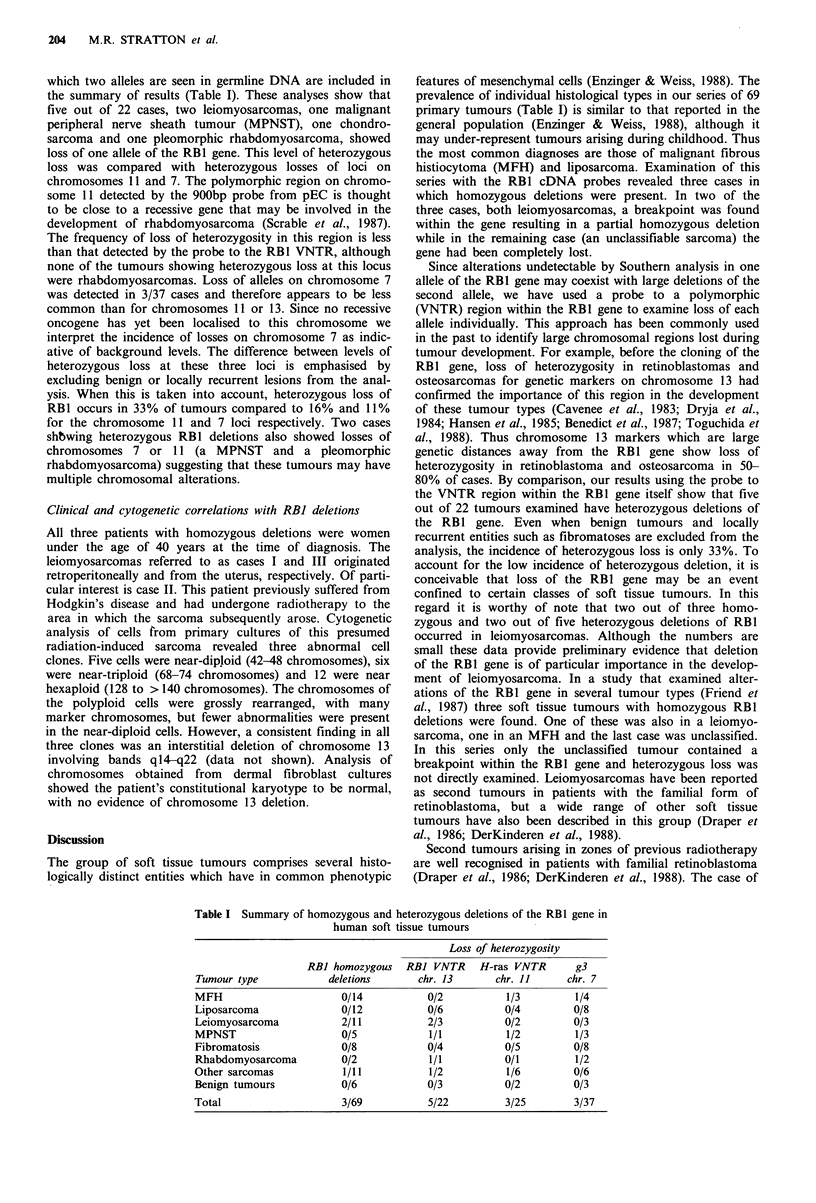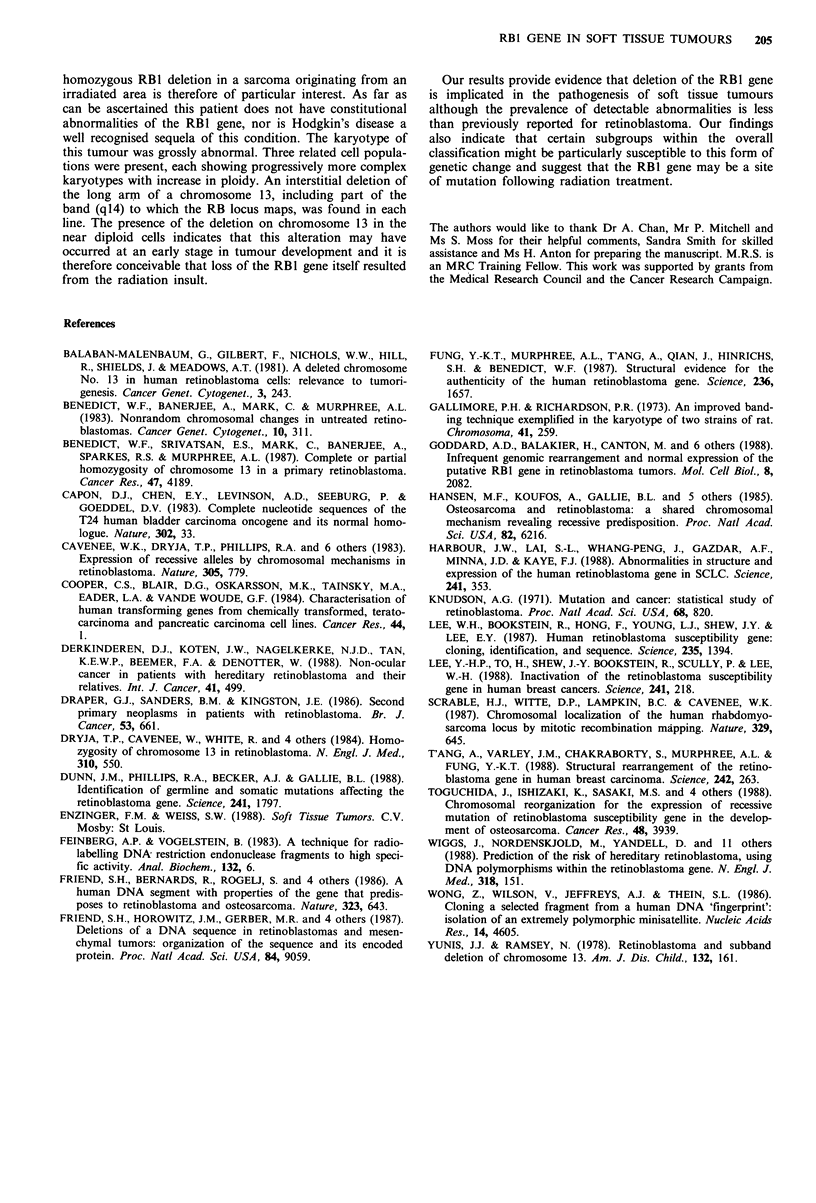# Structural alterations of the RB1 gene in human soft tissue tumours.

**DOI:** 10.1038/bjc.1989.251

**Published:** 1989-08

**Authors:** M. R. Stratton, S. Williams, C. Fisher, A. Ball, G. Westbury, B. A. Gusterson, C. D. Fletcher, J. C. Knight, Y. K. Fung, B. R. Reeves

**Affiliations:** Section of Chemical Carcinogenesis, Institute of Cancer Research, London, UK.

## Abstract

**Images:**


 © The Macmillan Press Ltd., 1989

### Structural alterations of the RB1 gene in human soft tissue tumours

M.R. Stratton, S. Williams, C. Fisher, A. Ball, G. Westbury, B.A. Gusterson, C.D.M. Fletcher[1], J.C. Knight, Y.-K. Fung[2], B.R. Reeves & C.S. Cooper

*Sections of Chemical Carcinogenesis and Pathology, Institute of Cancer Research, Fulham Road, London SW3 6JB and 15 Cotswold Road, Belmont, Sutton, Surrey SM2 5NG, UK; [1]Department of Histopathology, St Thomas' Hospital, Lambeth Palace Road, London SE1, UK; and [2]Departments of Pediatrics and Microbiology, USC School of Medicine, Division of Hematology-Oncology, Childrens Hospital of Los Angeles, Los Angeles, CA 90027, USA.*

**Summary** Sixty-nine primary soft tissue tumours were examined for alterations of the RB1 gene which has previously been implicated in the genesis of retinoblastoma. In three tumours loss of both alleles of this gene (homozygous deletion) was detected. Two of these, both leiomyosarcomas, contained a chromosomal breakpoint within the RB1 gene, while in the third tumour, a radiation induced sarcoma, complete deletion was observed. Using a probe that detects a polymorphic locus within the RB1 gene we found loss of only one allele (heterozygous deletion) in 33% of soft tissue sarcomas examined, including two leiomyosarcomas, a malignant peripheral nerve sheath tumour, a rhabdomyosarcoma and a chondrosarcoma. When taken together our results suggest that alterations of the RB1 locus may play an important part in the pathogenesis of soft tissue tumours and particularly in leiomyosarcomas which accounted for four of the eight RB1 alterations observed in this study.

In recent years recombinant DNA technology has allowed isolation and characterisation of some of the genes involved in tumour induction. Through study of transforming retroviruses and the use of the NIH3T3 mouse fibroblast transformation assay several dominantly acting oncogenes have been identified. These require an activating event in only one of the two alleles normally present in somatic cells to enable them to exert their transforming effect. By contrast there is a distinct group of recessive genes which require alterations of both alleles before they can contribute to tumour formation. In the latter group genetic alterations appear to result in loss of gene function and it is presumed that these genes normally act to suppress tumour formation. The paradigm for the recessive oncogenes is the gene at the RB locus on the long arm of chromosome 13, the loss of which has been implicated in the development of retinoblastoma.

Retinoblastoma is a paediatric embryonal tumour which may present in a familial or sporadic pattern. On the basis of the clinical and epidemiological characteristics of this tumour Knudson proposed a model of tumour induction requiring two hits or mutations (Knudson, 1971). Patients with the familial form of the disease carry one hit in the germ line while individuals who develop the tumour in a sporadic fashion acquire both hits by somatic mutation. Characterisation of germline and tumour cell karyotypes led to the suggestion that these two notional hits represent deletion or inactivation of both alleles of a gene on the long arm of chromosome 13 (Yunis & Ramsey, 1978; Balaban-Malenbaum *et al.*, 1981; Benedict *et al.*, 1983). Recently this gene (RB1) has been cloned as a 4.7kb cDNA (Friend *et al.*, 1986) and alterations in the form of deletions and point mutations have been detected in retinoblastoma specimens (Friend *et al.*, 1986, 1987; Fung *et al.*, 1987; Lee *et al.*, 1987; Goddard *et al.*, 1988; Dunn *et al.*, 1988).

The possibility that RB1 is involved in the development of other tumour types arises from the observation that patients with the familial form of the disease often develop second tumours several years after treatment of their retinoblastoma (Draper *et al.*, 1986; Friend *et al.*, 1987; DerKinderen *et al.*, 1988). These second tumours are most commonly osteosarcomas of bone but also include a variety of soft tissue tumours. Structural alterations of the RB1 gene have been detected in osteosarcomas (Friend *et al.*, 1986, 1987; Fung *et al.*, 1987) and, interestingly, in some tumour types not commonly associated with the retinoblastoma trait such as breast carcinoma (T'Ang *et al.*, 1988; Lee *et al.*, 1988) and small cell carcinoma of the lung (Harbour *et al.*, 1988). In the present study we have examined a large series of soft tissue tumours for homozygous alterations of RB1 and in addition have assessed the prevalence of heterozygous deletions. Our data suggest that alterations of the RB1 gene play a role in the development of certain types of soft tissue tumour.

### Methods

Primary tumours, peripheral bloods, muscle and skin biopsies were obtained from branches of the Royal Marsden Hospital, Surrey and London, and St Thomas' Hospital London. Sarcoma cell lines were obtained from the American Type Culture Collection. Southern analysis of DNA extracted from these specimens was performed on nylon hybridisation membranes using conventional protocols (Cooper *et al.*, 1984). Probes were radiolabelled using random primers (Feinberg & Vogelstein, 1983). The probes used were the 4.7kb RB1 cDNA (divided into 0.9kb and 3.8kb fragments at an internal EcoR1 site) (Fung *et al.*, 1987), p68RS2.0, a 2.0kb probe to the variable number of tandem repeat (VNTR) region of the RB1 gene from Dr T. Dryja (Wiggs *et al.*, 1988), the g3 fingerprinting probe which detects sequences on chromosome 7 from Cellmark Diagnostics courtesy of Prof. A. Jeffreys (Wong *et al.*, 1986) and 0.9kb Mspl fragment of the pEC plasmid acting as a probe to the VNTR region adjacent to the c-H-*ras* protooncogene on chromosome 11 (Capon *et al.*, 1983). For cytogenetic analysis, metaphases were Giemsa banded by conventional methods (Gallimore & Richardson, 1973).

### Results

### *Detection of homozygous deletions of the RB1 gene*

Primary soft tissue tumours and cell lines were examined by Southern analysis using fragments derived from the 4.7kb RB1 cDNA. The smaller of these, 0.9kb in size, detects 14.5, 5.8, 1.5 and 1.2kb HindIII bands while the 3.8kb probe detects 5.3, 4.5, 7.4, 9.4, 6.2 and 2.1kb HindIII bands on Southern blots of normal human DNA (Figures 1 and 2). Of the 69 primary tumours, three (two leiomyosarcomas and

Correspondence: M.R. Stratton.
Received 18 January 1989, and in revised form, 31 March 1989.

**Figure 1** Homozygous deletions in the RB1 gene detected on Southern blots of HindIII digested DNAs hybridised to 0.9kb (**a**) and 3.8kb (**b**) probes derived from the 4.7kb RB1 cDNA. DNAs are from: normal human leukocytes, lanes 1 and 2; case I, a leiomyosarcoma, lane 3; case II, an unclassifiable sarcoma, lane 4; case III, a leiomyosarcoma, lane 5.

one unclassifiable case) showed abnormal band patterns (Figure 1). In case I, a leiomyosarcoma, only the 14.5kb band detected by the 0.9kb RB1 probe remains. In case II, the unclassifiable tumour, the gene appears entirely deleted. Residual signal in this tumour is due to contamination of the specimen by a population of stromal cells estimated at approximately 15% of the total by densitometry. In case III, a leiomyosarcoma, the band pattern generated by the 0.9kb probe is normal but several bands usually detected by the 3.8kb probe are absent. The nature of the deletions which give rise to these abnormal band patterns is illustrated in Figure 2, where the genomic HindIII fragments detected by the RB1 probes are displayed according to their order on the 200kb of DNA spanning the RB1 gene. Two of the three

**Figure 2** Schematic illustration of homozygous deletions in the RB1 gene in the cases shown in Figure 1. HindIII fragments detected by the RB1 cDNAs are ordered along the 200kb of chromosomal DNA which contain the gene. The boxes representing the cDNA probes (not to scale) lie directly underneath HindIII fragments which they detect. The gaps in the solid lines representing DNAs from tumours I, II and III mark the extent of homozygous deletions.

deletions (both in leiomyosarcomas) terminate within the RB1 gene and extend 3′ to it. In case I the breakpoint lies between the 14.5kb and 1.2kb HindIII fragments while in case III it lies within the 3′ region of the gene. The 5′ extent of these deletions has been confirmed by using a probe to a region just 5′ to the first exon of the RB1 gene (data not shown). Interestingly, the locations of these breakpoints correspond to zones containing two large introns of the RB1 gene. None of the set of cell lines which included a leiomyosarcoma, a leiomyoblastoma, two rhabdomyosarcomas and a desmoid tumour showed abnormalities. The background smear and the intense band running just below the 14.5kb band on blots probed with the 0.9kb probe is apparently due to a GC rich region which hybridises to sequences outside the RB1 gene (Fung *et al.*, 1987).

### *Detection of heterozygous deletions of the RB1 gene*

Homozygous deletions detectable by Southern analysis using the RB1 cDNA probes probably constitute a small minority of the abnormalities in this gene. In particular alterations such as point mutations or small deletions will not be detectable by this approach. To investigate cases where a small alteration on one allele may coexist with a large deletion of the other, we have used a probe to a polymorphic (VNTR) region within the RB1 gene (Wiggs *et al.*, 1988), which allows each allele to be visualised independently (Figure 3).

In the cases illustrated two bands, each corresponding to one allele of the RB1 gene, are seen in germline DNA (Figure 3, lanes 1 and 3). However, in tumours from the respective patients (Figure 3, lanes 2 and 4) only one of these bands is seen. Hence these cases have lost at least part of one RB1 allele during tumour development. Only cases in

**Figure 3** Examples of the detection of heterozygous deletions of the RB1 gene. Southern blots of Rsa I digested DNAs hybridised to p68RS2.0 which detects the VNTR region within the RB1 gene. DNAs are from: germline and tumour tissue from a case of leiomyosarcoma, lanes 1 and 2 respectively; germline and tumour tissue from a case of pleomorphic rhabdomyosarcoma, lanes 3 and 4 respectively. The 2.0kb size marker is indicated.

which two alleles are seen in germline DNA are included in the summary of results (Table I). These analyses show that five out of 22 cases, two leiomyosarcomas, one malignant peripheral nerve sheath tumour (MPNST), one chondrosarcoma and one pleomorphic rhabdomyosarcoma, showed loss of one allele of the RB1 gene. This level of heterozygous loss was compared with heterozygous losses of loci on chromosomes 11 and 7. The polymorphic region on chromosome 11 detected by the 900bp probe from pEC is thought to be close to a recessive gene that may be involved in the development of rhabdomyosarcoma (Scrable *et al.*, 1987). The frequency of loss of heterozygosity in this region is less than that detected by the probe to the RB1 VNTR, although none of the tumours showing heterozygous loss at this locus were rhabdomyosarcomas. Loss of alleles on chromosome 7 was detected in 3/37 cases and therefore appears to be less common than for chromosomes 11 or 13. Since no recessive oncogene has yet been localised to this chromosome we interpret the incidence of losses on chromosome 7 as indicative of background levels. The difference between levels of heterozygous loss at these three loci is emphasised by excluding benign or locally recurrent lesions from the analysis. When this is taken into account, heterozygous loss of RB1 occurs in 33% of tumours compared to 16% and 11% for the chromosome 11 and 7 loci respectively. Two cases showing heterozygous RB1 deletions also showed losses of chromosomes 7 or 11 (a MPNST and a pleomorphic rhabdomyosarcoma) suggesting that these tumours may have multiple chromosomal alterations.

### *Clinical and cytogenetic correlations with RB1 deletions*

All three patients with homozygous deletions were women under the age of 40 years at the time of diagnosis. The leiomyosarcomas referred to as cases I and III originated retroperitoneally and from the uterus, respectively. Of particular interest is case II. This patient previously suffered from Hodgkin's disease and had undergone radiotherapy to the area in which the sarcoma subsequently arose. Cytogenetic analysis of cells from primary cultures of this presumed radiation-induced sarcoma revealed three abnormal cell clones. Five cells were near-diploid (42–48 chromosomes), six were near-triploid (68–74 chromosomes) and 12 were near hexaploid (128 to >140 chromosomes). The chromosomes of the polyploid cells were grossly rearranged, with many marker chromosomes, but fewer abnormalities were present in the near-diploid cells. However, a consistent finding in all three clones was an interstitial deletion of chromosome 13 involving bands q14–q22 (data not shown). Analysis of chromosomes obtained from dermal fibroblast cultures showed the patient's constitutional karyotype to be normal, with no evidence of chromosome 13 deletion.

### Discussion

The group of soft tissue tumours comprises several histologically distinct entities which have in common phenotypic features of mesenchymal cells (Enzinger & Weiss, 1988). The prevalence of individual histological types in our series of 69 primary tumours (Table I) is similar to that reported in the general population (Enzinger & Weiss, 1988), although it may under-represent tumours arising during childhood. Thus the most common diagnoses are those of malignant fibrous histiocytoma (MFH) and liposarcoma. Examination of this series with the RB1 cDNA probes revealed three cases in which homozygous deletions were present. In two of the three cases, both leiomyosarcomas, a breakpoint was found within the gene resulting in a partial homozygous deletion while in the remaining case (an unclassifiable sarcoma) the gene had been completely lost.

Since alterations undetectable by Southern analysis in one allele of the RB1 gene may coexist with large deletions of the second allele, we have used a probe to a polymorphic (VNTR) region within the RB1 gene to examine loss of each allele individually. This approach has been commonly used in the past to identify large chromosomal regions lost during tumour development. For example, before the cloning of the RB1 gene, loss of heterozygosity in retinoblastomas and osteosarcomas for genetic markers on chromosome 13 had confirmed the importance of this region in the development of these tumour types (Cavenee *et al.*, 1983; Dryja *et al.*, 1984; Hansen *et al.*, 1985; Benedict *et al.*, 1987; Toguchida *et al.*, 1988). Thus chromosome 13 markers which are large genetic distances away from the RB1 gene show loss of heterozygosity in retinoblastoma and osteosarcoma in 50–80% of cases. By comparison, our results using the probe to the VNTR region within the RB1 gene itself show that five out of 22 tumours examined have heterozygous deletions of the RB1 gene. Even when benign tumours and locally recurrent entities such as fibromatoses are excluded from the analysis, the incidence of heterozygous loss is only 33%. To account for the low incidence of heterozygous deletion, it is conceivable that loss of the RB1 gene may be an event confined to certain classes of soft tissue tumours. In this regard it is worthy of note that two out of three homozygous and two out of five heterozygous deletions of RB1 occurred in leiomyosarcomas. Although the numbers are small these data provide preliminary evidence that deletion of the RB1 gene is of particular importance in the development of leiomyosarcoma. In a study that examined alterations of the RB1 gene in several tumour types (Friend *et al.*, 1987) three soft tissue tumours with homozygous RB1 deletions were found. One of these was also in a leiomyosarcoma, one in an MFH and the last case was unclassified. In this series only the unclassified tumour contained a breakpoint within the RB1 gene and heterozygous loss was not directly examined. Leiomyosarcomas have been reported as second tumours in patients with the familial form of retinoblastoma, but a wide range of other soft tissue tumours have also been described in this group (Draper *et al.*, 1986; DerKinderen *et al.*, 1988).

Second tumours arising in zones of previous radiotherapy are well recognised in patients with familial retinoblastoma (Draper *et al.*, 1986; DerKinderen *et al.*, 1988). The case of

**Table I** Summary of homozygous and heterozygous deletions of the RB1 gene in human soft tissue tumours

| | | *Loss of heterozygosity* | | |
|---|---|---|---|---|
| *Tumour type* | *RB1 homozygous deletions* | *RB1 VNTR chr. 13* | *H-ras VNTR chr. 11* | *g3 chr. 7* |
| MFH | 0/14 | 0/2 | 1/3 | 1/4 |
| Liposarcoma | 0/12 | 0/6 | 0/4 | 0/8 |
| Leiomyosarcoma | 2/11 | 2/3 | 0/2 | 0/3 |
| MPNST | 0/5 | 1/1 | 1/2 | 1/3 |
| Fibromatosis | 0/8 | 0/4 | 0/5 | 0/8 |
| Rhabdomyosarcoma | 0/2 | 1/1 | 0/1 | 1/2 |
| Other sarcomas | 1/11 | 1/2 | 1/6 | 0/6 |
| Benign tumours | 0/6 | 0/3 | 0/2 | 0/3 |
| Total | 3/69 | 5/22 | 3/25 | 3/37 |

homozygous RB1 deletion in a sarcoma originating from an irradiated area is therefore of particular interest. As far as can be ascertained this patient does not have constitutional abnormalities of the RB1 gene, nor is Hodgkin's disease a well recognised sequela of this condition. The karyotype of this tumour was grossly abnormal. Three related cell populations were present, each showing progressively more complex karyotypes with increase in ploidy. An interstitial deletion of the long arm of a chromosome 13, including part of the band (q14) to which the RB locus maps, was found in each line. The presence of the deletion on chromosome 13 in the near diploid cells indicates that this alteration may have occurred at an early stage in tumour development and it is therefore conceivable that loss of the RB1 gene itself resulted from the radiation insult.

Our results provide evidence that deletion of the RB1 gene is implicated in the pathogenesis of soft tissue tumours although the prevalence of detectable abnormalities is less than previously reported for retinoblastoma. Our findings also indicate that certain subgroups within the overall classification might be particularly susceptible to this form of genetic change and suggest that the RB1 gene may be a site of mutation following radiation treatment.

The authors would like to thank Dr A. Chan, Mr P. Mitchell and Ms S. Moss for their helpful comments, Sandra Smith for skilled assistance and Ms H. Anton for preparing the manuscript. M.R.S. is an MRC Training Fellow. This work was supported by grants from the Medical Research Council and the Cancer Research Campaign.

### References

BALABAN-MALENBAUM, G., GILBERT, F., NICHOLS, W.W., HILL, R., SHIELDS, J. & MEADOWS, A.T. (1981). A deleted chromosome No. 13 in human retinoblastoma cells: relevance to tumorigenesis. *Cancer Genet. Cytogenet.*, **3**, 243.

BENEDICT, W.F., BANERJEE, A., MARK, C. & MURPHREE, A.L. (1983). Nonrandom chromosomal changes in untreated retinoblastomas. *Cancer Genet. Cytogenet.*, **10**, 311.

BENEDICT, W.F., SRIVATSAN, E.S., MARK, C., BANERJEE, A., SPARKES, R.S. & MURPHREE, A.L. (1987). Complete or partial homozygosity of chromosome 13 in a primary retinoblastoma. *Cancer Res.*, **47**, 4189.

CAPON, D.J., CHEN, E.Y., LEVINSON, A.D., SEEBURG, P. & GOEDDEL, D.V. (1983). Complete nucleotide sequences of the T24 human bladder carcinoma oncogene and its normal homologue. *Nature*, **302**, 33.

CAVENEE, W.K., DRYJA, T.P., PHILLIPS, R.A. and 6 others (1983). Expression of recessive alleles by chromosomal mechanisms in retinoblastoma. *Nature*, **305**, 779.

COOPER, C.S., BLAIR, D.G., OSKARSSON, M.K., TAINSKY, M.A., EADER, L.A. & VANDE WOUDE, G.F. (1984). Characterisation of human transforming genes from chemically transformed, teratocarcinoma and pancreatic carcinoma cell lines. *Cancer Res.*, **44**, 1.

DERKINDEREN, D.J., KOTEN, J.W., NAGELKERKE, N.J.D., TAN, K.E.W.P., BEEMER, F.A. & DENOTTER, W. (1988). Non-ocular cancer in patients with hereditary retinoblastoma and their relatives. *Int. J. Cancer*, **41**, 499.

DRAPER, G.J., SANDERS, B.M. & KINGSTON, J.E. (1986). Second primary neoplasms in patients with retinoblastoma. *Br. J. Cancer*, **53**, 661.

DRYJA, T.P., CAVENEE, W., WHITE, R. and 4 others (1984). Homozygosity of chromosome 13 in retinoblastoma. *N. Engl. J. Med.*, **310**, 550.

DUNN, J.M., PHILLIPS, R.A., BECKER, A.J. & GALLIE, B.L. (1988). Identification of germline and somatic mutations affecting the retinoblastoma gene. *Science*, **241**, 1797.

ENZINGER, F.M. & WEISS, S.W. (1988). *Soft Tissue Tumors*. C.V. Mosby: St Louis.

FEINBERG, A.P. & VOGELSTEIN, B. (1983). A technique for radiolabelling DNA restriction endonuclease fragments to high specific activity. *Anal. Biochem.*, **132**, 6.

FRIEND, S.H., BERNARDS, R., ROGELJ, S. and 4 others (1986). A human DNA segment with properties of the gene that predisposes to retinoblastoma and osteosarcoma. *Nature*, **323**, 643.

FRIEND, S.H., HOROWITZ, J.M., GERBER, M.R. and 4 others (1987). Deletions of a DNA sequence in retinoblastomas and mesenchymal tumors: organization of the sequence and its encoded protein. *Proc. Natl Acad. Sci. USA*, **84**, 9059.

FUNG, Y.-K.T., MURPHREE, A.L., T'ANG, A., QIAN, J., HINRICHS, S.H. & BENEDICT, W.F. (1987). Structural evidence for the authenticity of the human retinoblastoma gene. *Science*, **236**, 1657.

GALLIMORE, P.H. & RICHARDSON, P.R. (1973). An improved banding technique exemplified in the karyotype of two strains of rat. *Chromosoma*, **41**, 259.

GODDARD, A.D., BALAKIER, H., CANTON, M. and 6 others (1988). Infrequent genomic rearrangement and normal expression of the putative RB1 gene in retinoblastoma tumors. *Mol. Cell Biol.*, **8**, 2082.

HANSEN, M.F., KOUFOS, A., GALLIE, B.L. and 5 others (1985). Osteosarcoma and retinoblastoma: a shared chromosomal mechanism revealing recessive predisposition. *Proc. Natl Acad. Sci. USA*, **82**, 6216.

HARBOUR, J.W., LAI, S.-L., WHANG-PENG, J., GAZDAR, A.F., MINNA, J.D. & KAYE, F.J. (1988). Abnormalities in structure and expression of the human retinoblastoma gene in SCLC. *Science*, **241**, 353.

KNUDSON, A.G. (1971). Mutation and cancer: statistical study of retinoblastoma. *Proc. Natl Acad. Sci. USA*, **68**, 820.

LEE, W.H., BOOKSTEIN, R., HONG, F., YOUNG, L.J., SHEW, J.Y. & LEE, E.Y. (1987). Human retinoblastoma susceptibility gene: cloning, identification, and sequence. *Science*, **235**, 1394.

LEE, Y.-H.P., TO, H., SHEW, J.-Y. BOOKSTEIN, R., SCULLY, P. & LEE, W.-H. (1988). Inactivation of the retinoblastoma susceptibility gene in human breast cancers. *Science*, **241**, 218.

SCRABLE, H.J., WITTE, D.P., LAMPKIN, B.C. & CAVENEE, W.K. (1987). Chromosomal localization of the human rhabdomyosarcoma locus by mitotic recombination mapping. *Nature*, **329**, 645.

T'ANG, A., VARLEY, J.M., CHAKRABORTY, S., MURPHREE, A.L. & FUNG, Y.-K.T. (1988). Structural rearrangement of the retinoblastoma gene in human breast carcinoma. *Science*, **242**, 263.

TOGUCHIDA, J., ISHIZAKI, K., SASAKI, M.S. and 4 others (1988). Chromosomal reorganization for the expression of recessive mutation of retinoblastoma susceptibility gene in the development of osteosarcoma. *Cancer Res.*, **48**, 3939.

WIGGS, J., NORDENSKJOLD, M., YANDELL, D. and 11 others (1988). Prediction of the risk of hereditary retinoblastoma, using DNA polymorphisms within the retinoblastoma gene. *N. Engl. J. Med.*, **318**, 151.

WONG, Z., WILSON, V., JEFFREYS, A.J. & THEIN, S.L. (1986). Cloning a selected fragment from a human DNA 'fingerprint': isolation of an extremely polymorphic minisatellite. *Nucleic Acids Res.*, **14**, 4605.

YUNIS, J.J. & RAMSEY, N. (1978). Retinoblastoma and subband deletion of chromosome 13. *Am. J. Dis. Child.*, **132**, 161.